# Evaluation of the Influence of Build Orientation on the Surface Roughness and Flexural Strength of 3D-Printed Denture Base Resin and Its Comparison with CAD-CAM Milled Denture Base Resin

**DOI:** 10.1055/s-0043-1768972

**Published:** 2023-06-09

**Authors:** Naji Ahmad Alharethi

**Affiliations:** 1 Department of Prosthodontic Sciences, College of Dentistry in Ar Rass, Qassim University, Saudi Arabia

**Keywords:** 3D printing, CAD-CAM, flexural strength, surface roughness

## Abstract

**Objectives** The purpose of this study was to determine the surface roughness and flexural strength of a three-dimensional (3D)-printed denture base resin printed with two different build plate orientations and to compare them with a computer-aided design-computer-aided manufacture (CAD-CAM) milled denture base resin.

**Materials and Methods** Sixty-six specimens ( *n* = 22/group) were prepared by 3D printing and CAD-CAM technology. The group A and B specimens were 3D-printed bar-shaped denture base specimens printed at 120-degree and 135-degree build orientation, respectively, whereas group C specimens were milled using a CAD-CAM technology. The surface roughness was assessed using a noncontact profilometer with a 0.01 mm resolution and the flexural strength was determined using a three-point bend test. The maximum load in Newtons (N) at fracture, the flexural stress (MPa), and strain (mm/mm) was also measured.

**Statistical Analysis** Data were analyzed by a statistical software package. One-way analysis of variance test was applied to determine whether significant differences existed among the study groups, followed by Bonferroni post-hoc test to determine which resin group significantly differed from the others in terms of flexural strength and surface roughness ( *p* ≤ 0.05).

**Results** The flexural stress (MPa) of group C was 200% of group A and 166% of group B. The flexural modulus was 192% of group A and 161% of group B. In contrast, group A had the lowest mean value among the three groups for all the parameters. No significant difference was seen between group A and group B. The mean roughness values of the CAD-CAM denture base resin specimens (group C) were the least (127356 nm) among all the three groups. The mean surface roughness of the 3D-printed denture base specimens (group A) was 1,34,234 nm and that of group B was (1,45,931 nm); however, it was statistically nonsignificant ( *p* > 0.05)

**Conclusions** The CAD-CAM resin displayed superior surface and mechanical properties compared to the 3D-printed resin. The two different build plate angles did not have any significant effect on the surface roughness of the 3D-printed denture base resin.

## Introduction

For many years, heat-cured poly-methyl-methacrylate was the material of choice for denture base materials. It is simple to produce, inexpensive, and has several favorable physicomechanical properties. Unfortunately, it has a high polymerization shrinkage, low elastic modulus, impact strength, fatigue resistance, and flexural strength, leading to fracture. 1 2 3

The likelihood of fracture from poor fatigue resistance arises from the stress that accumulates over time in parts of the material where fractures emerge because of applied pressure, particularly chewing forces. 1 4 5 6 When a prosthesis breaks by impact, it usually happens when the patient takes out their prosthesis to clean it and it slips out of their hands. 7 8

The computer-aided design and computer-aided manufacture (CAD-CAM) of dental restorations is becoming more and more common because of recent developments in digital technology. 1 9 10 Subtractive or additive methods of fabricating dental restorations using CAD-CAM technology 10 11 12 13 can solve various shortcomings of conventional techniques. 8 11 13 14

Additive manufacturing, commonly referred to as three-dimensional (3D) printing or rapid prototyping, uses layer by layer deposition of material to create an object from a 3D model. 11 12 14 In contrast, subtractive manufacturing uses a computer numeric controlled machine to mill the dental restoration in multiple axes from a block or disc of material. Digital technologies provide the advantage of rapid denture manufacturing and fewer stages in the workflow, which can lessen the likelihood of errors. 9 10 15 While milling is commonly used to make digital dentures, 3D printing denture bases has several benefits. It is less expensive, does not require the use of rotary tools, produces minimal wastage, and has the capability to generate several objects at the same time. 11 12

The surface roughness of the materials used as a denture base is a critical property and should be kept within acceptable values to avoid plaque accumulation, bacterial colonization, and staining. 16 17 Although avoiding surface roughness might be a complicated task in digital manufacturing due to the nature of the object production, 17 18 studies have found that surfaces with roughness values higher than 0.2 μm maximize the rate of bacterial colonization. 17 19 20 This roughness is a normal sequel to the layer-by-layer building of the object in 3D printing technology. 3 4 However, some printing parameters may influence the object's accuracy as well as surface smoothness. 17 18 Among these parameters, build orientation is an important factor that should be considered. Several researchers have documented changing the build orientation to manipulate the geometry and improve the surface details and smoothness. 21 22 Various studies focused on accuracy, materials consumption, and time of processing of digital manufacturing technique, which should match high strength and surface smoothness as well. 23 24

A Medline search revealed few research studied on the influence of the digital manufacturing and build orientation on the flexural strength of denture base resins. To the best of our knowledge, there are no studies evaluating the influence of build orientations of 120 and 135-degree build angles on the surface roughness and flexural strength of 3D-printed denture base resins. Thus, the aim of this study was to evaluate the impact of two build orientations of a 3D-printed denture base resin on its surface roughness and flexural strength and to compare it with those of a CAD-CAM manufactured denture base resin. The null hypothesis was that no difference would be found between the flexural strength and surface roughness of the 3D-printed denture base resin specimen groups and the CAD-CAM manufactured precured denture base specimen group or between the 3D-printed denture base groups built with two different build plate orientation.

## Materials and Methods

Based on the study design three groups were planned, (group A) 3D-printed at a 120-degree build orientation, (group B) 3D-printed at a 135-degree build orientation and (group C) milled using a CAD-CAM machine. The specimen size was calculated using power analysis software (G*Power v3.1.9.4; Heinrich-Heine-Universitat Dusseldorf, Germany) (Total specimen size = 66; effect size [f] = 0.5; actual power = 95%; power (1-ẞ err prob) = 95%; α = 0.05). Based on the calculation each group had 22 specimens.

**Fig. 1:**
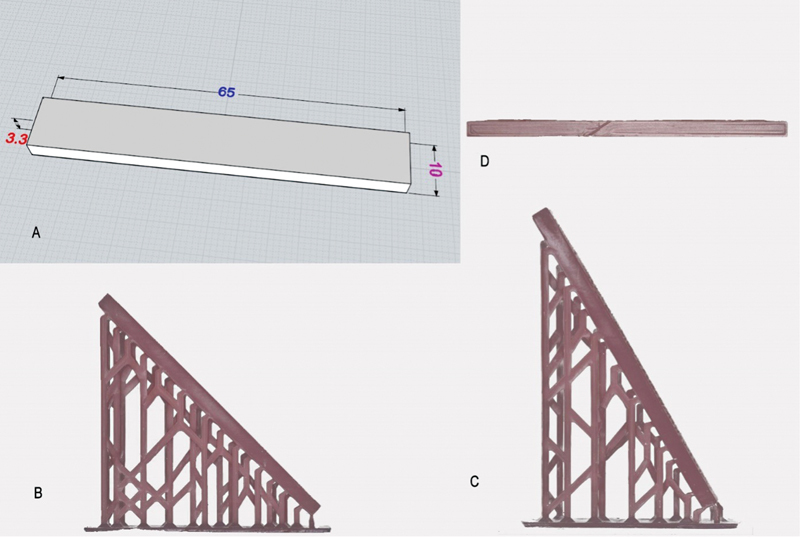
( **A** ) Specimen designed in computer-aided design designing software using documented dimensions. ( **B** and **C** ) Three-dimensional printed specimens built at 135 and 120 degrees, respectively. ( **D** ) Specimen milled by computer-aided design-computer-aided manufacture machine.

Three-dimensionally bar-shaped specimen (65 × 10 × 3.3 mm) were virtually designed in CAD software (MOI v 3, Triple Squid Software Design, United States) to prepare the specimens ( Fig. 1 ) for the three-point bend test based on the International Organization for Standardization (ISO) standard. 7 11 The STL file of the virtual design was exported into the ASIGA Composer software (ASIGA Composer v 1.1.7).

The 3D-printed groups were printed by a digital light processing ASIGA Max 3D printer (Asiga MAXTM; ASIGA, Sydney, Australia) at 50 μm layer thickness from a photosensitive resin (ASIGA DentaBase; ASIGA, Sydney, Australia). The 3D printing processing software was customized to print objects and their support structures at 120 and 135 degrees at UV energy equal to 385 nm with a pixel resolution of 62 µm and light-emitting diode with the printing speed set to 50 mm/h. After 3D printing, the specimens were washed in isopropyl alcohol for 10 minutes and then dried. The specimens were subjected to post-processing curing for 20 minutes in an Asiga Flash post-curing chamber (ASIGA, Sydney, Australia) following which all the supports were removed. 25

The printed specimens were measured (65 × 10 × 3.3 mm) first by the same operator to ensure standardization and then finished using silicon carbide (mega-Schmirgelleinen; megadental, Büdingen, Germany) followed by acrylic polishing burs (Shofu Dental Corporation, San Marcos, California, United States), pumice (Kemdent Works, Wiltshire, United Kingdom) and high shine polishing compound (Keystone Industries, New Jersey, United States).

The CAD-CAM specimens (group C) were manufactured from the same 3D designed model with precured denture base resin discs (IvoBase CAD; Ivoclar Vivadent AG) in a CAD-CAM machine (Ceramill Motion 2; Amann Girrbach, Austria; Fig. 1D ). All the specimens were collected and marked to ease identification. The specimens were then stored in distilled water for 30 days (37 ± 1°C) to mimic the plasticizing effect experienced in the oral cavity 26

### Surface Roughness Testing

Specimen surface roughness (Ra) was recorded using a noncontact profilometer (Contour GT-K1 optical profiler; Bruker Nano GmbH, Berlin, Germany) at a resolution of 0.01 μm and a total measurement length of 0.8 mm. Surface roughness was measured at four different areas on each polished specimen and was repeated a total of three times. The average value of the surface roughness (µm) was calculated for each specimen. The generated images were processed by specialized software (Vision64; Bruker Nano GmbH, Berlin, Germany) to analyze the pit features.

### Flexural Strength Testing

At the time of testing the specimens were removed from the storage box, cleaned, and dried. The specimen was then placed with its ends on the two supports of the testing machine (Model LRX; Lloyds Instruments Ltd, United Kingdom) at a fixed 50 mm distance. The load was then applied at a constant displacement rate of 1 mm/minute, a preload of 1.0 N, and a preload speed of 10 mm/minute until fracture occurred. The maximum load in Newtons (N) at fracture was recorded as well as the flexural stress (MPa), strain (mm/mm), and its modulus (MPa) were calculated and plotted by the machine software based on the equations below. 7 11

Flexural strength = 3FL/2bh ^2^ (1)

elastic modulus = FL ^3^ /4bh ^3^ d (2)

Where, FS is the flexural strength (MPa), F is the load or force at which fracture occurred (N), L is the span of specimen between the supports, b is the width, and d is the thickness of the specimen.

### Statistical Analysis

The data were analyzed by a statistical software package (SPSS Statistics, version 21.0, IBM). The homogeneity of variance and normal distribution were analyzed by Levene's and Kolmogorov–Smirnov tests, respectively. Accordingly, one-way analysis of variance test was applied to determine whether significant differences existed among the study groups, followed by Bonferroni post-hoc test to determine which resin group significantly differed from the others in terms of flexural strength and surface roughness at *p* -value less than or equal to 0.05.

## Results

**Fig. 2:**
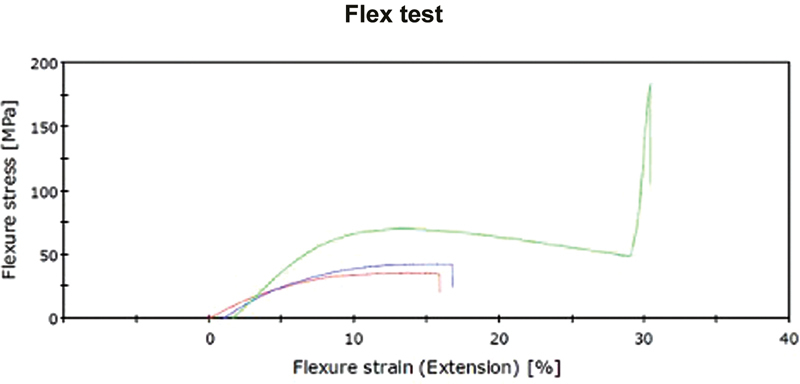
Flexural stress (MPa)—Strain (%) curve plotted for the three groups. Group A is in red, while groups B and C are represented by blue and green colors, respectively.

Data collected from the flexural strength test showed statistically significant higher values for the CAD-CAM milled denture base resin (group C) than for the 3D-printed denture base resin groups (group A and B) for all the calculated parameters. The flexural stress (MPa) of group C was 200% of group A and 166% of group B. The flexural modulus was 192% of group A and 161% of group B. In contrast, group A had the lowest mean value among the three groups for all the parameters. No significant difference was seen between group A and group B ( Table 1 and Fig. 2 ).

**Table 1:** Comparison of the mean and standard deviation of the surface roughness test, and the flexural strength test results between all tested groups

Groups	Maximum load (N)	Flexure stress at yield (MPa)	Flexure strain (mm/mm)	Flexure modulus (MPa)
Group A	71.76 ± 4.25 ^a*^	35.881 ± 3.72 ^a^	0.1589 ± 0.0081 ^a^	577.16 ± 26.54 ^a^
Group B	85.47 ± 6.10 ^a^	42.734 ± 4.44 ^a^	0.1598 ± 0.012 ^a^	687.00 ± 32.31 ^a^
Group C	365.47 ± 12.42 ^b^	70.263 ± 7.21 ^b^	0.28754 ± 0.04 ^b^	1109.61 ± 57.91 ^b^
ANOVA**	*F* = 1180; *p* < 0.001	*F* = 34.84; *p* < 0.001	*F* = 27.24, *p* = 0.001	*F* = 139.40; *p* < 0.001

Abbreviation: ANOVA, analysis of variance.

* Groups with different letters in the same column are statistically different, *p* ≤ 0.05. **The Fisher value and *p* -value for ANOVA test.

**Fig. 3:**
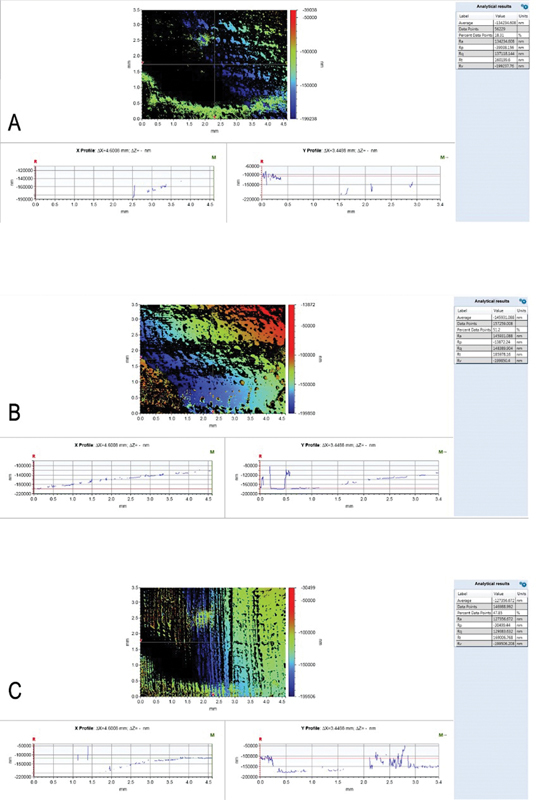
Surface roughness analysis of the three groups. Each image represents the color-coded scale of the surface topology (nm) while demonstrating the X and Y profile changes in (nm) throughout the studied surface. ( **A** and **B** ) three-dimensional printed denture base specimens printed at 120- and 135-degree angles, respectively, while ( **C** ) is the surface roughness of milled computer-aided design-computer-aided manufacture group specimen.

The mean roughness values of the CAD-CAM denture base resin specimens (Group C) were the least (127356 nm) among all the three groups. The mean surface roughness of the 3D-printed denture base specimens (group A) was 1,34,234 nm and that of group B was (1,45,931 nm); however, it was statistically nonsignificant ( *p* > 0.05) ( Fig. 3 ). There was a statistically significant difference between the surface roughness of the group C specimens and the group A ( *p* = 0.031) and group B specimens ( *p* = 0.01).

## Discussion

This study assessed the flexural strength and surface roughness of digitally manufactured denture base materials using subtractive (CAD-CAM) and additive technology (3D-printed) built in two different orientations. The results showed a statistically higher flexural strength and surface roughness values for the CAD-CAM denture base resin when compared to the 3D-printed groups, while no significant difference was found between the two-3D-printed denture base resin groups printed at different build orientations. Accordingly, the null hypothesis was partially rejected.

The flexural strength and the flexural modulus of the CAD-CAM denture base group were significantly higher than both of the 3D-printed denture base resin groups. Similarly, improved mechanical properties were also reported by Al-Dwairi et al, for CAD-CAM denture base resin material. 27 These findings could be attributed to the characteristics of the manufacturing technology. The flexural strength values obtained in our research could be explained based on the internal structural geometry of the two materials. The 3D-printed acrylic resins used for the processing of complete dentures have a lower double-bond conversion, which directly impacts their mechanical properties. Certainly, the precured CAD-CAM discs have a better chance of curing in industrial plants, which enhances the fusion between polymer chains and cross-linking agents. This process improves material structure and minimizes the chances of porosity and crack propagation. This material is used for engraving and cutting in multiple axes by accurate milling machines to form the required shape of the object in a controlled environment. 5 9 On the other hand, 3D printing forms the object in a layer-by-layer process, which makes the object vulnerable to void formation and incomplete fusion of the particles that could happen in the post-curing chamber. 9 18 Although the 3D-printed sample groups had lower flexural strength values when compared to CAD-CAM manufactured group, they still met the ISO requirements for flexural strength (65 MPa) 3

On the other hand, the effect of the build angle was not effective in flexural strength enhancement. The build angles of 120 and 130 degrees were selected based on the results of previously published literature on best build angles for prosthesis accuracy. 28 29 The difference between the angles selected might be insufficient to demonstrate a significant change in flexural strength. Apparently, to produce denture bases, the 3D printing technology uses unpolymerized liquid resins, and once polymerized, an extra final light polymerization step is imperative to avoid distortion. Although 3D printing technology offers various advantages, such as precision, less material waste, and lower infrastructure costs, 10 incomplete polymerization before the final light-polymerization stage can result in polymerization shrinkage and reduced strength. Deformation of the prosthesis may occur when removing the partially polymerized specimen from the platform. Furthermore, a residual layer of unpolymerized resin is usually present on the finished prosthesis and must be completely cleaned with a suitable solvent. 22 23

The surface roughness of the CAD-CAM denture base resin group was significantly lower than the 3D-printed denture base groups. These findings are in agreement with findings of Helal et al *,* who also found that milled denture base resins had a lower roughness values compared to 3D-printed and polyamide resins. 8 The surface roughness generated on the resin surface followed the type of the manufacturing process used. For example, when the 3D printing technology is used, the specimen created is in a layer-by-layer increments creating minute step like structured surface. This type of surface topography is unavoidable but can be highly controlled by minimizing the layer thickness, using proper printing orientation and technique. Based on the results of our study, the 120-degree angle demonstrated less surface roughness than the 135-degree angle, which confirms the influence of the build orientation. 24 25 However, the difference between them was not statistically significant to recommend one build orientation over the other. These findings could be attributed to the simplicity of the specimen shape configuration used in this study, and may reflect different results if an actual denture print was to be considered. 22

On the other hand, CAD-CAM denture base specimens are created by subtraction from pre-manufactured discs using a milling machine. The burs cut the object from the disc layer by layer until the full form is achieved. There is no doubt that milling flat surface specimens is simple and produces a smoother surface compared to 3D printing if proper machine settings were selected. 15 Another issue in the 3D printing process that could be source of roughness is the possibility of partially cured particle formation especially if adequate post-curing is not achieved. These particles may dislodge and form minute porosities. Fortunately, this is a nonissue in CAD-CAM manufacturing technology as the premanufactured discs are already fully polymerized. 9 10

This study has some limitations that can be addressed by future research studies. These include evaluating the effect of dynamic loading, thermocycling, water sorption, fracture toughness, color stability, and biocompatibility. Testing different resin materials available in the market and using different post-curing polymerization cycles may also show interesting results.

## Conclusion

Based on the findings of our *in vitro* original research study, statistically significant differences in flexural strength and surface roughness were found between the CAD-CAM denture base resin group and the two-3D-printed denture base resin groups printed at 120 and 130 degrees, respectively. The CAD-CAM resin displayed superior surface and mechanical properties compared to the 3D-printed resin. The two different build plate angles did not have any significant effect on the surface roughness of the 3D-printed denture base resin.

## Notes

Appendix- ANOVA-Flexural strength raw data
**ANOVA for maximum load (N)**

**ANOVA for flexure stress at yield (MPa)**

**ANOVA for flexure strain (mm/mm)**

**ANOVA for flexure modulus (MPa)**
